# Transperitoneal Multiport Robotic Partial Nephrectomy for Hilar Tumors: Step by Step

**DOI:** 10.5152/tud.2025.25019

**Published:** 2025-07-29

**Authors:** Indraneel Banerjee, Jared Robinson, Indrait Banerjee

**Affiliations:** 1 Department of Urology and Robotic Surgery, Penn Highlands Healthcare Standard Institution, Pennsylvania, United States; 2 Department of Surgery, Sir Seewoosagur Ramgoolam Medical College Standard Institution, Vacoas-Phoenix, Plaines Wilhems District – Mauritius; 3 Department of Pharmacology, Sir Seewoosagur Ramgoolam Medical College Standard Institution, Vacoas-Phoenix, United States

Robotic partial nephrectomy for hilar renal tumors is a complex surgery. As compared to non-hilar renal masses, these tumors have equivalent oncological outcomes but have an increased risk of complications.^1^ In this video, we will discuss the step-by-step technique for achieving an optimal surgical outcome in multiport transperitoneal robotic partial nephrectomy for renal hilar tumors. Two selected cases of robotic partial nephrectomy for hilar tumors performed by a single surgeon (IB) in a single institution were chosen specifically for their illustrative value. These cases demonstrate key surgical principles and decision-making strategies rather than aiming to represent a comprehensive or generalizable series. The intent is educational, highlighting critical steps and considerations in complex renal tumor management.

A 71-year-old female presented with a 6.7 × 5.5 cm right posterior renal hilar mass with baseline Cr 0.83. In a posterior hilar tumor, the kidney needs to be mobilized entirely and flipped so that the lower pole becomes anterior and the upper pole becomes posterior, to facilitate easier dissection.^2^ Nephropexy is also required at completion in posterior hilar tumors, as the kidney needs to be mobilized extensively. The second patient is a 68-year-old male with a 3.5 × 3.5 cm right anterior renal hilar mass with baseline Cr 1.16. In anterior hilar tumors, the kidney should not be mobilized extensively, as the tumor will tend to fall toward the hilum, making dissection difficult. Moreover, careful dissection of the ureter is warranted. For both locations, preoperative planning with proper imaging studies, intraoperative ultrasound, intra-hilar dissection, enucleoresection technique, clipping or repairing branch arteries, and early unclamping was performed.^3^ Postoperative follow-up CT scan revealed well-perfused kidneys without any complications.

## Results

One consideration when using the polar flip technique for posterior hilar tumors is the presence of multiple renal arteries that branch early. In such cases, the distance between the main renal artery and its branches may restrict the rotation of the kidney. Therefore, these posterior hilar tumors may be more effectively approached through the retroperitoneal route.^4^ However, the majority of urologists worldwide still prefer the transperitoneal approach for robotic partial nephrectomy. Thus, the surgical approach demonstrated in the video remains relevant.

To conclude, the video illustrates the surgical technique of robotic partial nephrectomy for hilar tumors to enhance both oncological and functional outcomes.

Informed consent was obtained from all patients featured in this video article. Patients were made aware of the purpose, procedures, potential risks, and benefits of the video, and their participation was entirely voluntary. The confidentiality and anonymity of patient data were maintained in accordance with ethical guidelines.

Institutional Review Board approval was *waived*, as this educational video article involved only de-identified surgical footage and did not include any prospective research or patient interventions beyond standard clinical care.

## Figures and Tables

**Table:** 

	Case 1 (Posterior Hilar)	Case 2 (Anterior Hilar)
Warm ischemia time	21 minutes	30 minutes
Blood loss	200 mL	100 mL
Length of stay	2 days	1 day
Histopathology	Clear cell RCC Grade 2, pT1b, Margins negative	Clear cell RCC Grade 1, pT1a, Margins negative
Post operative Cr	0.9	1.16
Duration of follow-up	13 months	13 months
